# *Nigella sativa* and Thymoquinone: A Natural Blessing for Periodontal Therapy

**DOI:** 10.3390/antiox9121260

**Published:** 2020-12-11

**Authors:** Mohamed Mekhemar, Yasmine Hassan, Christof Dörfer

**Affiliations:** Clinic for Conservative Dentistry and Periodontology, School of Dental Medicine, Christian-Albrecht’s University, 24105 Kiel, Germany; yasminemounir87@gmail.com (Y.H.); doerfer@konspar.uni-kiel.de (C.D.)

**Keywords:** thymoquinone, *Nigella sativa*, periodontal disease, periodontitis, anti-inflammatories, antioxidants, adjunctive periodontal therapy, herbal remedies

## Abstract

Thymoquinone (TQ), the chief active constituent of *Nigella sativa* (NS), shows very valuable biomedical properties such as antioxidant, antimicrobial, anticancer, anti-inflammatory, antihypertensive, hypoglycemic, antiparasitic and anti-asthmatic effects. Several studies have examined the pharmacological actions of TQ in the treatment of oral diseases but its potential role in periodontal therapy and regeneration is not yet fully defined. The present investigation has been designed to review the scientific studies about the effects of TQ as an adjunct to periodontal treatment to promote healing and periodontal regeneration. Along with clinical experiments, in vitro studies exhibit the beneficial effects of TQ during periodontal therapy. Nevertheless, additional comprehensive clinical and preclinical studies at cellular and molecular levels are essential to examine the particular action mechanisms of *Nigella sativa* and its elements, particularly TQ, during periodontal treatment or regeneration.

## 1. Introduction

Recently, worldwide attention on the use of therapeutic herbs in the treatment of different diseases has been growing extensively owing to their promising outcomes and rare side effects [1]. As explained by the World Health Organization (WHO), more than two-thirds of the world’s general population, mainly in developing nations, depend on the use of natural remedies and traditional herbs for their main health care and disease treatment. Thus, the WHO has encouraged emerging countries to integrate the use of therapeutic plants as a supplementary resource to increase the effectiveness of the health care programs [1,2]. Among the evidence-based herbal remedies, highly ranked as a “miracle herb” is *Nigella sativa* (NS) [3,4] NS is a yearly blossoming plant from the family *Ranunculaceae* also known as black cumin, black seed, habbatul barakah, black caraway, kalojeera, kalonji or kalanji and is native to the southern regions of Asia and numerous countries in the Middle East and the Mediterranean region [4,5,6]. In historic and traditional medicine, NS has been utilized for centuries for the treatment of numerous illnesses of the body and psyche. The medicinal assets of NS have been established in the Islamic medicine, Chinese traditional treatments, Unani, Ayurveda and other medicinal systems [7,8]. Investigations have accredited the health benefits of NS due to its rich nutritional content and active components [4]. The seeds are composed of fixed and essential oils, alkaloids, proteins and saponins [4,9]. Among the pharmacologically active components that have been isolated from NS up to now the most described active and therapeutic constituent is thymoquinone (TQ) [9]. Numerous investigators have expansively reviewed NS and TQ and found them to have various medicinal activities, such as anti-inflammatory, antioxidant, antimicrobial, analgesic, histamine release inhibitory, hypoglycemic, anticarcinogenic, antihypertensive, immune-activating and hepatoprotective effects [3,4].

Infections of the periodontal ligament and gingiva have plagued humans since early historic ages. Paleo-pathological reports have displayed that periodontal complications specified by bone loss have pathologically affected early humans in diverse cultures around the world [10]. gingivitis is the slightest form of periodontal disease and defines the gingival inflammation due to the accumulation of plaque and bacteria between the gumline and tooth. This reactive condition is reversible with improving oral hygiene. In periodontitis the periodontal condition develops into a chronic, irreversible, bone destroying inflammatory disease. This induces a host immune response to protect against the invading microorganisms. During the defensive mechanism against the bacteria, the host immune reactions also incite the periodontal destruction through the loss of bone attachments, subsequently leading to alveolar bone loss of the affected tooth [11]. Non-surgical handling can be effective in mild to moderate periodontitis while surgical action is important in progressive conditions [10]. Adjunctive to the mechanical scaling and root planning in the treatment of periodontal diseases, confined systems of drug delivery, mouth washes, irrigations and devices for sustained drug release are used regularly for conveyance of therapeutic agents [10,12]. Furthermore, it has been reported that regular periodontal treatment along with the use of an adjunctive therapeutic mediator can improve patient outcomes significantly compared to mechanical treatment alone [10,13]. Modern day chemotherapeutic mediators exhibited significant effectiveness in the treatment of periodontal disease, but display a number of objectionable adverse effects such as taste alteration, tooth and tongue discoloration, antimicrobial resistance and the higher prices of these compounds [14,15]. Thus, the application of natural and herbal products as NS and TQ for periodontal treatment has garnered increased attention recently and could provide great benefits, especially to populations of lower socioeconomic status around the globe [4,16]. 

In order to explore the topic of *Nigella sativa* and thymoquinone application during periodontal therapy and their mode of action, the search terms thymoquinone, *Nigella sativa*, black seed, black caraway, black cumin, kalojeera, kalonji, or kalanji in combination with periodontal disease, periodontal regeneration, periodontal therapy, periodontal treatment, or periodontitis were entered into PubMed, MEDLINE, Google Scholar and the Cochrane database. The hierarchical system of evidence-based medicine was then applied within the review. Laboratory-based experiments, in vivo animal studies and clinical investigations were identified and reported in the review. This investigation was not planned to be a systematic review and consequently recommendations were not made as such. As more research is needed currently in this area, this article tries to use the best available evidence to demonstrate the scientific status of the topic and the need for more studies to expand the current knowledge.

## 2. Periodontitis and Periodontal Therapy

### 2.1. Periodontitis as a Global Health Problem

Periodontitis can be described as an advanced destruction of the tooth-supporting structures of the oral cavity, including alveolar bone, cementum and periodontal ligaments. Its main action is revealed via loss of clinical periodontal attachments and radiographically measured loss of alveolar bone, leading to a clinical indication of periodontal pockets, gingival inflammation and bleeding. If periodontal disease remains untreated, it can terminate with tooth loss, however this condition is treatable and preventable if correct therapy is applied [17]. Owing to its high occurrence worldwide, periodontal disease presents a foremost problem of public health and health-related quality of life, as it can lead to tooth loss, masticatory and verbal disabilities, as well as esthetic and psychological complications. Periodontitis-related edentulism further accounts for significant dental care costs and presents a serious financial problem facing a large proportion of people worldwide [18]. As a chronic inflammatory and multifactorial condition linked to dental plaque biofilm dysbiosis, periodontitis is considered the most shared chronic inflammatory disease of all human societies. As explained by previous studies, the worldwide age-standardized occurrence of severe periodontitis in the last decade was nearly 11%, promoting periodontitis to be almost among the five most widespread conditions globally [19], while other investigations described the prevalence of mild periodontal disease as high as 50% worldwide [20]. If periodontitis remains untreated, or is not sufficiently handled medically, it can further become accountable for many years of disability and malfunction longer than all other human diseases [21]. Moreover, periodontal diseases are linked with a wide spectrum of systemic conditions that can lead to death or other severe health incapacities, such as cardiovascular diseases [22], diabetic complications [23] and various adverse consequences during pregnancy [24]. On an international economic scale, periodontal disease is reported to cost more than $50 billion and additional $25 billion in direct and indirect treatment costs, respectively [21]. As periodontitis-linked edentulism plays a major role in the financial burden of dental diseases due to the replacement of lost teeth it causes significant increases in the total economic weight related to dentistry. The overall cost of dental conditions, was estimated recently to be of $544.41 billion with $356.80 billion direct expenses, as well as $187.61 billion indirect expenses [25]. 

### 2.2. Strategies for the Treatment of Periodontal Disease

#### 2.2.1. Mechanical Therapy

Dental plaque encompasses a combination of microorganisms, minerals and food deposits. As some plaque may harden into dental calculus, or may remain in hard reachable areas of the dentition, both dental deposits might not easily be removed by toothbrushing or flossing and can cause gingival inflammation, as a primary stage that develops afterwards to periodontal disease [11]. The most effective procedure for inhibition of this destructive process of inflammation is considered periodontal scaling. Through the measures of dental scaling, periodontists aim to remove soft and hard deposits, as well as stains from the crown and root surfaces of affected teeth [26]. Scaling and root planning has developed to become the benchmark of nonsurgical mechanical treatment of periodontitis. Numerous clinical trials have designated that it diminishes the bacterial level inside periodontal pockets significantly and recovers clinical outcomes as levels of clinical attachment, bleeding on probing and probing depths [27].

Scaling and root planning can be completed by different periodontal instruments, such as hand scalers and curettes, or sonic and ultrasonic tools that enhance the competence of the periodontist to reach into dental root furcations and through the pocket depths effectively [26]. During the localized therapy of scaling and root planning it is not always possible to eliminate all pathogenic microorganisms due to their existence within periodontal tissues, or in very deep pockets that might be hard to reach by instruments. This led to the introduction of the adjunctive combination of antimicrobial and chemotherapeutic agents beside the main mechanical periodontal debridement to improve the microbiological profile and control the periodontal inflammation and regeneration [28,29]. Other advanced techniques, as laser treatments are also being investigated for periodontal therapy. Features as the ability to excise, evaporate and sterilize periodontal pockets allow the laser treatment to be applied as an adjunct or alternative to regular mechanical periodontal treatment. Nevertheless, more research has to be performed to investigate the bacterial recolonization after laser therapy in periodontal disease [26]. 

#### 2.2.2. Chemotherapeutic Periodontal Therapy

##### Host Modulation Therapy (HMT)

Previous clinical and histological observations of periodontal disease have shown that periodontal tissue deterioration is initiated by microbiological plaque and bacterial toxins, besides the important factor of the host immune and inflammatory response to the pathogens [30]. This major immune aspect of periodontal disease pathogenesis inspired the introduction of host modulatory therapy (HMT) to modify the host immune reaction and minimize the immune mediated destruction levels [31]. Several host modulatory mediators are available for chemotherapeutic periodontal treatment, adjunctive to the mechanical debridement by localized or systemic administration, for example doxycycline at sub-antimicrobial dose [32], anti-inflammatory drugs, including non-steroidal anti-inflammatory drugs (NSAID), steroids, anti-IL1 and anti-TNF [33], bisphosphonates, various growth factors [34] and enamel matrix derivatives [35]. These chemotherapeutic mediators share the ability to modulate the host-immune reaction by different mechanisms, eventually blocking the destructive characteristics of the inflammation [31]. Such mechanisms include the inhibition of prostaglandins and pro-inflammatory cytokines by anti-inflammatory agents, the suppression of collagenase by tetracyclines and doxycycline, as well as the reduced osteoclast cell activity mediated through bisphosphonates [26]. 

##### Antimicrobial Therapy

As mechanical debridement of periodontal pockets and tissues is not always efficient enough to eliminate all microbes, residual bacteria in the periodontal environment can often recolonize the tissues after several weeks of therapy [36]. Consequently, adjunctive application of systemic chemotherapeutic antimicrobial agents besides the mechanical and surgical therapy has proven to be more effective in the complete eradication of the pathogens [37]. Although systemic administered antimicrobial therapy has revealed many positive effects in the periodontal treatment [38,39], it is generally prescribed only for rapidly progressing or refractory periodontitis due to its major simultaneous disadvantages [26]. Such drawbacks include for instance the unpredictable concentration of the antibiotic at the targeted site, a potential rapid decrease in plasma antibiotic concentration below the needed therapeutic index and a possible resistance development by the microorganisms against antibiotics [26,39]. Furthermore, prescribing high doses of systemic antibiotics may cause various side effects in a large number of patients [26,39]. These mentioned potential disadvantages of systemic administered antimicrobial chemotherapeutics endorsed the development and investigation of localized intra-pocket drug delivery systems for periodontal therapy [29]. This localized drug delivery inside the periodontal pockets displayed less drug-related adverse effects, higher concentrations of the drug at the targeted site for extended periods of time and lead to better patient compliance [26]. The local drug delivery, required for antibacterial agents, as well as other types of chemotherapeutic mediators in periodontal treatment, can be administered by a localized application of therapeutic oral gels and solutions, or by an insertion of special delivery devices into the periodontal pockets, such as periodontal chips for a sustained release of the needed drug concentration facilitated by the gingival crevicular fluid [26,29,37]. 

##### Herbal Therapeutic Agents

Herbal remedies and compounds encompass natural plant elements acknowledged traditionally and scientifically to have therapeutic benefits [10]. Owing to their promising results and fewer side effects compared to systemic drugs and other chemotherapeutic agents, global interest in the use of natural herbs in various therapeutic procedures has been growing widely [10]. Corresponding to this attention to herbal remedies along with a wide spectrum of treatments, periodontal treatments recently introduced the application of different herbal chemotherapeutic agents adjunctive to scaling and root planning [10,40]. This aimed to avoid the various adverse outcomes of modern chemical therapeutics, including the mentioned side effects of antibiotics, as well as tooth and tongue discolorations [41], taste alteration [41] and the financial burden caused by the high costs of the drugs [10]. The herbs and natural products often used for periodontal treatment include *Acacia catechu*, *Cinnamomum zeylanicum*, *Propolis*, *Mikania laevigata*, *Mikania glomerate*, *Glycyrrhiza glabra*, *Aloe vera*, *Droserapeltata*, *Allium sativum*, *Helichrysumitalicum*, *Azadirachta indica*, *Coptidis rhizome*, *Piper cubeba*, *Azadirachta indica*, *Syzygium aromaticum*, tea tree oil and *Salvadora persica* [1,10]. In addition to the mentioned herbal preparations a wide spectrum of herbal based compounds is currently undergoing clinical trials and have shown potential therapeutic benefits. Among the most prominent herbal remedies with noticeable budding future benefits for periodontal treatment is NS, as an extensively used herb in traditional medicine in the Middle East and Asia for the treatment of a wide array of illnesses and conditions [1]. 

## 3. *Nigella sativa* and Thymoquinone as Therapeutic Compounds

### 3.1. Cultural and Historical Importance of Nigella sativa

Among many herbal remedies that have been described in different regions of the world, NS is a well-established cultural and religion-based medication for various health conditions [42]. Being a plant native to the southern European continent, North Africa, the Middle East, as well as being widely cultivated in areas around the Indian Peninsula, NS became a vastly used herbal remedy in many cultures and households [42]. Furthermore, migration throughout history assisted to further spread NS cultivation into other European regions and the American Continent [42]. Linguistically, NS seeds are known by different names of several language origins due to its wide cultural impact around the globe. Black seed is one of the well-known names as the seeds turn black when exposed to air [43]. Amongst the Muslim and Arabic community, NS is denoted as alhabattul sawdaa (black seed), or habbatul barakah (blessing seed) [44]. In other parts of the world, it is also known as black caraway, black cumin, kalojeera, kalonji, shuniz, or kalanji [45]. In different folklore traditions and many civilizations, NS has a long history of recognition as a “miracle herb” due to its capability to treat different conditions and supports the natural healing process of the body [4]. Even ancient civilizations thousands of years BC, such as the ancient Egyptian, or Ptolemaic cultures have known NS for its “magical” effects and reserved it at their religious locations [46]. One of the earliest inscribed texts about NS can be found in the book of Isaiah of the Bible, naming the herb as “Ketzah” in Hebrew and describing it as a spice used for baking [42]. For members of Muslim societies, the use of NS and its oils in their traditional Islamic herbal medicine is chiefly based on an authentic prophetic declaration that NS is a cure of all diseases, except death; as quoted in the works of prominent Muslim scholars [47]. This foundation promoted the status of *NS* among Muslim scholars and scientists throughout history and inspired them to devise studies and medical treatments embracing NS and its oils. The Persian physician Ibn Sina, known in western countries as Avicenna, discussed NS in his well-known medical treatise “*Canon of Medicine*”. In this text, which was considered a key medical transcript worldwide until the 17th century, he specified that NS has protective and healing properties and aids in complete body recovery. He further commended NS as a medication against numerous diseases, such as fever, colds and for wound healing and regeneration [48]. Starting in the last decades of the 20th century and up to the present time numerous investigations continued to assess the therapeutic features of NS and its bioactive constituent TQ in diverse medical fields and described the potential roles TQ and NS might play in the future within clinical therapies or disease preventing measures [42]. 

### 3.2. Bioactive Compounds of Nigella sativa Seeds

NS oil has been studied during different historical eras in medicine and as confirmed by recent day investigations to be the most effective component of the NS plant. This efficacy is mainly ascribed to the quinone elements of the NS oil, especially TQ, identified as the most significant bioactive compound that makes up almost 50% of all NS oil constituents [42]. Additional functional constituents of the *NS* oil include carvacrol, *p*-cymene, dihydrothymoquinone, thymohydroquinone, thymol, *t*-anethole, *α*-thujene, *α*-pinene, *β*-pinene and *γ*-terpinene. TQ exclusively displays the main medicinal effects of NS, such as strong anti-inflammatory, antioxidant, antimicrobial, analgesic, histamine release inhibitory, hypoglycemic, anticarcinogenic, antihypertensive, immune-activating, hepatoprotective effects and anti-diabetic properties [49]. 

### 3.3. Sources of Nigella sativa Oil and Thymoquinone

NS seeds are the core natural source containing TQ compounds [50]. Besides the *Ranunculaceae* plant family, considered a main source of NS seeds, other types of the *Lamiaceae* family such as *Coridothymus, Agastache, Monarda, Origanum, Mosla, Satureja,* and *Thymus* were also described as possible sources for TQ extraction [50]. Similarly, chemical isolation of TQ has also been reported from *Tetraclinis* plants, as well as the *Cupressaceae* family [51]. Reduced forms of TQ like dithymoquinone and thymohydroquinone have also been detected in trace amounts in other plant families and may share similar medicinal characteristics with TQ [52]. 

### 3.4. Pharmacological Properties of Nigella sativa and Thymoquinone

#### 3.4.1. Chemical Composition and Structure

The chemical composition of *Nigella sativa* is diverse. The seeds encompass protein (26.7%), fat (28.5%), carbohydrates (24.9%), crude fibre (8.4%) and total ash (4.8 %). Among these components, several therapeutically active compounds and elements have been isolated and reported previously [3,53] (Table 1).

**Figure 1:**
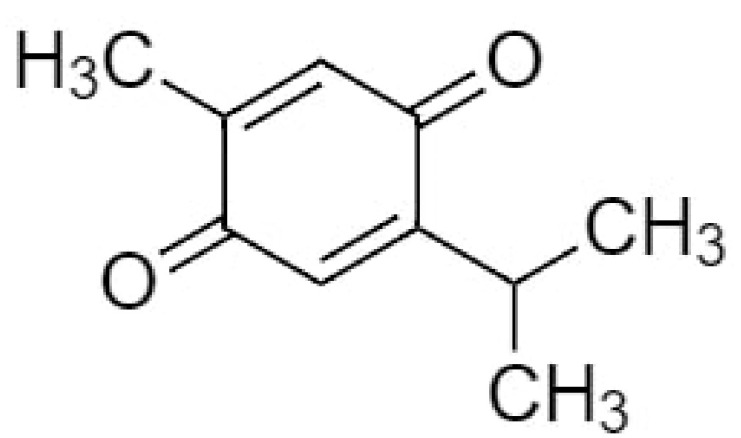
Chemical structure of thymoquinone.

TQ (2-isopropyl-5-methylbenzo-1,4-quinone), the most effective bioactive component of NS seeds has the molecular formula C_10_H_12_O_2_ and a molar mass of 164.20 g/mol [54] (Figure 1). It is a 10-carbon compound having a basic quinone ring moiety of six carbons with the 7th methyl group carbon at position C2 and the 8th, 9th and 10th carbon propyl group at position C5. The compound contains two carbon–carbon double bonds and two carbon-oxygen double bonds. It has a 0 hydrogen bond donor count, whereas its hydrogen bond acceptor count is 2 and the rotatable bond count is 1, with the log*p* value of 2. The topological polar surface area of TQ is 34.1 A^2^ [55].

The TQ concentration in the *Nigella sativa* oil has been described to be between 18 and 25 µg/mL. In thermogravimetric analysis the thermal decomposition of TQ initiates at 65 °C and ends at 213 °C. Previous studies have reported that TQ is a member of the monoterpene class of natural complexes and displays keto-enol tautomerism [56]. Specifically, the keto configuration is the main arrangement involved in the therapeutic effects of TQ [57]. TQ solubility varies in the range of 549–669 µg/mL in aqueous solutions [58]. Moreover, TQ is mostly unstable in aqueous solutions, predominantly at alkaline pH values and possess severe light sensitivity [56]. 

The chemical biosynthesis of TQ as a member of monoterpene compounds initiates in most species by cyclization of geranyl diphosphate and formation of *γ*-terpinene, that afterwards is aromatized into *p*-cymene and hydroxylated to carvacrol. Further hydroxylation of carvacrol occurs and allows the formation of thymohydroquinone. Thymohydroquinone then starts an oxidation process that finally leads to TQ formation [56]. 

#### 3.4.2. Routes of Administration and Drug Concentration

The drug formulation and bioavailability of TQ is restricted by its hydrophobic characteristics [59]. Administration of TQ can occur through several routes, including systemic intraperitoneal, intravenous, oral subacute and sub-chronic administration, besides the localized drug delivery of TQ [6,60,61]. In the current investigation the reviewed in vivo studies reported the local periodontal application of 0.1–0.2% TQ gel [62,63,64] and similarly in the form of fabricated periodontal chips containing 2.5 mg of TQ [65]. One animal investigation reported systemic TQ administration by gastric feeding, at a rate of 10 mg/kg/d [66]. In vitro studies on TQ or *NS* extract effects on periodontal diseases and bacteria administered solutions of 2% *NS* extract [63] and 0.01–10% concentrations of TQ [67,68]. All tested concentrations and routes of drug delivery showed significant periodontal health-favoring outcomes in terms of clinical periodontal parameters as plaque index (PI), gingival index (GI), probing pocket depth (PPD), bleeding on probing (BOP), clinical attachment levels (CAL) [62,65,66], as well as molecular parameters as gingival crevicular fluid alkaline phosphatase levels (GCF-ALP) [66] and cytokine/chemokine levels [63]. Microbiological and histological parameters similarly showed significant improvements as observed in biofilm formation and inflammatory cell infiltration (ICI) [66,67,68,69]. 

#### 3.4.3. Toxicological Profile of Thymoquinone

Various studies have investigated acute, subacute and, teratogenic and reproductive toxicities of TQ and various adverse effects of *NS* and its active constituents [70] (Table 2). 

In one of the investigations the LD_50_ value of TQ was observed to be 10 mg/kg after intraperitoneal injection in rats, while the same route of administration in mice at doses of 4, 8, 12.5, 25 and 50 mg/kg exhibited no effects on biochemical indices, such as lactate dehydrogenase or serum alanine transaminase. Nevertheless, intraperitoneal injections of TQ in mice with doses higher than 50 mg/kg had lethal outcomes [71]. In case of oral TQ administration, different toxicological investigations designated that doses of 10–100 mg/kg showed no toxic or adverse effects in mice [71,72,73].The maximum tolerated dose of intraperitoneal injected TQ in rats was 22.5 mg/kg in males and 15 mg/kg in females, while oral administration of TQ in both genders exhibited a higher maximum tolerated dose of 250 mg/kg [74]. This discrepancy in toxicity response between both routes of TQ administration can be attributed to a complete TQ absorption into the blood circulation after intraperitoneal injection, while in the oral route of administration, TQ is first bio-transformed in the gastrointestinal tract and metabolized in the liver [71]. In terms of potential genotoxicity and effects on embryonic development, it was also reported that TQ could possibly disrupt the embryonic development during the second trimester in rats and might cause chromosomal aberrations if its concentrations exceeded 80 mg/kg [70]. Further toxicity studies concluded that treatment with TQ can significantly induce oxidative effects, reduce cellular glutathione (GSH), elevate malondialdehyde (MDA) production and catalase (CAT) activity and also trigger p53 in a concentration and time-dependent way in various types of cells [70]. 

Overall, based on the performed studies, it could be determined that TQ and NS are mostly safe compounds which have a human history of thousands of years with no significant safety issues. Yet, to fulfil the worldwide regulatory safety standards it is important to perform more safety studies in the future to completely prepare these compounds and establish safety guidelines for application by each route of administration in clinical therapy [70].

#### 3.4.4. Development of Analogues of Thymoquinone

Several investigations have explored the development of TQ analog compounds with notable efficacy for different types of diseases [50]. Recently, new analogues of TQ were examined with sesquiterepene, monoterpenes and cytotoxic terpenes and indicated significant anti-cancer therapeutic activity [50,75]. Further studies revealed the promising properties of newly synthesized TQ analogs against radiation-induced dyslipidemia [76]. These analogues possessed the ability to inhibit (HMG-CoA) reductase and stimulate each of plasma LPL and LCAT enzymes concomitantly. Although some investigations presented a more prominent therapeutic effect of the synthetic mediators than natural TQ, traditionally extracted *NS* and TQ compounds seem to be less cytotoxic and have less adverse effects than their counterparts [50].

## 4. Modes of Action of *Nigella sativa* and Thymoquinone as Potential Adjuncts during Periodontal Therapy and in Periodontitis-Associated Settings

The effects of *Nigella sativa* extracts and thymoquinone in periodontal disease-associated settings and their modes of action with potential benefits for periodontal therapy have been investigated in several studies. These include in vitro studies, in vivo (animal) studies and clinical studies (RCTs). Relevant experiments to this investigation’s topic are listed in Table 3. The reported medicinal modes of action and the procedures and results of these inquiries are further explained in the following segment. 

### 4.1. Antioxidant and Anti-Inflammatory Effects

Inflammation and production of reactive oxygen species (ROS) have been reported as major players in the pathogenesis of periodontal disease [77]. In numerous investigations, NS and TQ have been discussed as anti-inflammatory [78] and antioxidant mediators with curative effects [79,80]. Previous studies explained that TQ induces an antioxidant effect through the scavenging ability of various free radicals, being as effective scavenging superoxide anions analogous to superoxide dismutase [81,82]. TQ has also been proven experimentally to show noticeable anti-inflammatory functions [78]. It reduces the levels of nitric oxide (NO) via reduction of iNOS mRNA production by macrophages and suppresses pro-inflammatory cytokines as IL-1b, IL-6, TNF-α, IFN-c and PGE2, simultaneous with an increase of the anti-inflammatory IL-10 [82,83]. The possible mechanism by which TQ employs this combined anti-inflammatory and anti-oxidant action might be associated to its aptitude to impede eicosanoid production. TQ and NS extracts have experimentally shown significant suppression of lipid peroxidation and eicosanoid generation, specifically, thromboxane B and leukotrienes B4, via inhibiting COX and LOX molecular pathways [84]. Considering these potential properties anti-inflammatory and anti-oxidant properties of TQ it may perform significant function to prevent the initiation and progression of periodontal disease as observed in the current review.

In the animal investigation proposed in one of the in vivo studies an assessment of the possible protective role of TQ on periodontal and gingival inflammation was performed [64]. The outcomes displayed that rats treated with TQ in their drinking water or as an oral gel had significantly lower values of periodontal indices and inflammatory clinical parameters in comparison to the control group. Histological investigation of their mandibular tissues correspondingly demonstrated no inflammatory signs in contrary to their controls. Another rat periodontitis model reported the preventive role of TQ in the initiation and progression of periodontal inflammation [66]. Rats’ molars gingival margins were ligated with a 4/0 silk suture to initiate periodontal inflammation. The trial involved 24 rats randomly and equally scattered into non-ligated, ligature only and ligature with TQ treatment (10 mg/kg gastric feeding) groups. After 11 days, the rats were sacrificed to examine the alveolar bone levels of the molars clinically and histologically.

**Figure 2:**
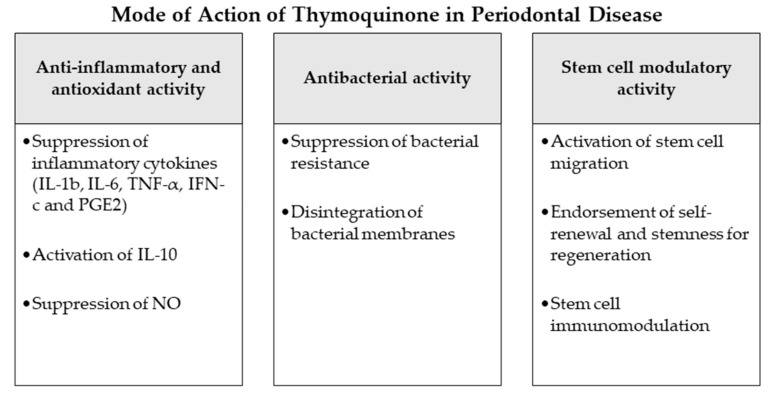
Mode of action of thymoquinone as an adjunctive periodontal chemotherapeutic in periodontal disease. *Abbreviations*: IL-1b: interleukin 1b, IL-6: interleukin 6, TNF-*α*: tumor-necrosis-factor-*α*, IFN-c: interferon c, PGE2: prostaglandin E2, IL-10: interleukin 10, NO: nitric oxide.

Statistically significant higher bone losses were observed in the ligature-only group in comparison with the two other groups. On a histological basis, the same outcome was reported concerning the ratio of ICI to osteoclasts. Furthermore, osteoblasts presented lower activity in the ligature only group in comparison with the other groups. Consequently, the outcome concluded that oral administration of TQ assisted in the prevention and suppression of periodontal inflammation and reduced alveolar bone resorption. Moreover, a clinical randomized single-blind split-mouth trial [65] evaluated the efficiency of a periodontal chip impregnated with 2.5 mg TQ in periodontal treatment. Twelve male patients with periodontal pockets were distributed into three different groups: control (no treatment), plain chitosan-periodontal chips, and TQ-impregnated chips. Compared to the control and plain periodontal chip group the TQ treated patients displayed significant improvements in the clinical attachments and other clinical periodontal parameters. This result concluded the advice to use of TQ chips as an adjunctive periodontal treatment besides the scaling and root planning and for maintenance visits. Recently, further clinical studies were performed on periodontitis patients to evaluate the potential benefits of local application of thymoquinone gel as an adjunctive to regular periodontal treatment. One of these investigations selected 20 patients with 40 test sites and divided them into two groups. The first group was treated by scaling and root planning in addition to local application of 0.2% TQ gel, while patients of the second group were only treated by regular scaling and root planning [62]. After 6 weeks a statistically significant decrease was detected in PPD and GCF-ALP levels besides a rise in periodontal attachment levels in the TQ group in contrast to the regular periodontal therapy patients. Another similar trial was completed on 48 periodontitis patients with application of 0.1% TQ gel adjunctive to the regular scaling and root planning [63]. Analogous to the reported previous outcomes, the TQ-treated group of this clinical study also presented a significant improvement in all clinical and biochemical parameters including PI, GI PPD and CAL, along with the of IL-1*β* inflammatory levels and total antioxidant capacity (TAOC) in the gingival crevicular fluid compared to the non-TQ-treated patients (Figure 2 and Table 3). 

### 4.2. Antibacterial Effects

Periodontal disease is a multifactorial pathophysiological process described by an inflammation of periodontal host tissues mediated by the immune response and linked to dysbiotic plaque biofilms, consequently destroying the tooth-supporting apparatus progressively and allowing the loss of periodontal attachment [17,85]. Following a gingival inflammation initiated by bacterial biofilm formation, dysbiotic ecological changes in the microbiome arise, responding to inflammatory and tissue breakdown products and immune responses and leading to the activation of several key molecular pathways promoting periodontal destruction. This mechanism finally activates host-derived proteases enabling the loss of marginal periodontal ligament fibers, junctional epithelium apical migration, concomitant with an apical spread of the microbial biofilm along the root surface [17,85]. Bacterial species of the identified “red complex” (*Treponema denticola*, *Porphyromonas gingivalis* and *Tannerella forsythia*) have been perceived historically as the chief infective microorganisms related to periodontitis [86]. This idea was nevertheless determined using culture-grounded investigations, which did not explore much of the microbiological diversity existing in samples [87]. Newer techniques have furthermore discovered other organisms also closely associated with periodontal disease such as the bacterial classes *Negativicutes*, *Erysipelotrichia* and *Clostridia* [88]; the genera Prevotella, Fusobacterium [89] and Synergistes [90]; as well as the species *Filifactor alocis* [88], *Methanobrevibacter oralis*, *Methanobacterium curvum/congolense*, *Methanosarcina mazeii* [87,91,92] and *Aggregatibacter actinomycetemcomitans* [93]. 

Multiple studies have investigated the antimicrobial effect of NS and TQ on different bacterial strains and species [50]. In numerous investigations TQ and NS compounds have displayed a significant bactericidal activity against Gram-negative and Gram-positive bacteria and inhibited bacterial biofilm formation [62,94], encouraging the application of TQ as an antimicrobial agent in diverse diseases [7,9]. One of the suggested mechanisms of TQ-mediated bactericidal ability was the targeted ROS generation against bacterial cells [94] with a simultaneous antioxidant and scavenging effect protecting the host tissues from oxidative stress [79]. Other studies observed a TQ-weakening effect on the integrity of bacterial membranes through pump efflux inhibition [95], which might also play an important role decreasing the bacterial resistance if TQ treatment was combined with antibiotics [96]. In the current investigation the reviewed studies reported in vivo and in vitro application of TQ and *NS* extract on different species of periodontitis-associated bacteria. In two animal studies, investigators applied 0.2% TQ as oral gel [62,64] or systemically [64] on subgingival bacteria in rat gingivitis and periodontitis models. On microbiological assessment, TQ administration was observed in both studies to be sensitive against *Porphyromonas gingivalis*, *A. actinomycetemcomitans* and *Prevotella intermedia* and could decrease the number of subgingival bacteria significantly [62,64]. Recently, conforming in vitro studies tested the effect of TQ [67,68] and *NS* [69] on the growth kinetics and activity [69], biofilm formation [67,68] and virulence [68] of periodontal-related bacterial species. Administration of 10% TQ showed significant effects on *Fusobacterium nucleatum* and *Porphyromonas gingivalis*, as results showed inhibition of biofilm formation and hemolysis activities of both bacteria [68]. TQ likewise repressed H_2_S production which is highly related with oral halitosis and disrupted bacterial membranes while reducing the expression of major virulence factors in both bacterial species [68]. TQ application in 0.1% or 0.01% solutions equally affected the biofilm formation of *Fusobacterium nucleatum* in a further investigation, as it indicated significant reduction of biofilm thickness and demonstrated a bacterial cleansing effect through TQ activity [67]. In another in vitro evaluation of *NS* extract antimicrobial properties, several microbial strains were isolated from the oral cavities of periodontitis patients [69]. The antimicrobial activities of *NS* were tested against *Staphylococcus epidermidis*, *Staphylococcus aureus*, *Streptococcus pneumoniae*, *Enterococcus faecalis*, *Klebsiella pneumoniae*, *Proteus* sp., *Acinetobacter baumannii/ calcoaceticus*, *Porphyromonas* sp. and *Veillonella* sp. [69]. The analysis attained by the microdilution method displayed high antibacterial activities of the essential oil extract of *NS* against all the observed species and mostly against *Staphylococcus epidermidis* and *Porphyromonas* sp. Nevertheless, the agar well diffusion method did not reflect the exact results, proving that numerous factors may impact the antimicrobial action of *NS* extracts [69] (Figure 2 and Table 3). 

### 4.3. Potential Stem Cell Modulation

The goal of periodontal therapy is to resist the microbial infection and rearrange the configurations and functions of the periodontium [97]. Difficult tasks persist in regenerating the periodontal structures and constructing bone-PDL-cementum complex concurrently [98]. Osteogenesis slightly precedes the cementum and periodontal ligament fibers differentiation. Subsequently, the oriented periodontal ligament fibers require to be attached to novel generated alveolar bone and cementum tissue, which is considered one of the most challenging processes in periodontal regeneration [99]. In recent years, numerous investigators discussed the high potential of periodontal regeneration with noticeable clinical success through mechanisms of regeneration for bone, cementum and PDL by mobilization of endogenous stem cells from their niches or transplanting exogenous stem cells targeting the periodontal defects [99]. Numerous mesenchymal stem cell (MSC) types persist and are accountable for homeostasis of the tissues, serving as a spring of renewable stem/progenitor cells to produce other essential cells throughout adult life [100]. Consequently, a successful periodontal regeneration depends on recruitment of locally-derived stem/progenitor cells, such as populations of resident periodontal or oral-tissue stem/progenitor cells, to the defect site for tissue homeostasis and succeeding differentiation into bone, periodontal ligament and cementum-forming cells [99,101]. Several studies have observed the modulating effects of natural herbs and compounds on the immune functions, migration, proliferation, cell fate determination and self-renewal abilities or “stemness” of various types of mesenchymal stem cells [102,103]. Among these studies, TQ and NS have displayed various effects that might influence the periodontal therapy and play a role in better regeneration and healing [104,105,106,107]. As explained in the investigations [104], TQ could activate c-MET and CXCR4 signaling pathways, promoting MSCs migration. TQ was correspondingly able to expand MSC immunomodulatory potential, as well as self-renewal ability and “stemness” by influencing the associated gene expression in vivo and in vitro [104,105]. Other experiments observed the significantly increased therapeutic potential and healing ability of MSCs after administration of NS extract to the damaged sites [106,108]. Such results may indicate a great regenerative accelerating potential of NS and TQ mediators if administered adjunctively to periodontal therapy. Nevertheless, more in vitro and in vivo investigations are needed, especially on periodontal ligament stem cells as major residents of periodontal defects to confirm and expand the knowledge about these potential advantages of NS and TQ (Figure 2 and Table 3). 

## 5. Conclusions

Recent years have witnessed a growth in herbal medicine inquiries to replace conservative treatments or use them as supportive medications for numerous diseases [1]. Diseases of oral and dental structures have not been an exception. Investigators have performed numerous experiments that examined the function of NS and TQ as its main active constituent in different areas of dental science [1,62,65,66,68,105]. NS and TQ display abundant potential therapeutic properties on diverse oral conditions. Besides its anticariogenic effects [1], NS and its constituent TQ play a significant role in the prevention and treatment of periodontal diseases as previewed in the current evaluation [62,63,64,65,66,67,68,69]. Frequent examinations have evaluated the sensitivity of periodontal and oral pathogens against NS extracts and TQ, and displayed outcomes equal to or enhanced than the antibiotics regularly used during treatment or regular periodontal treatment only [67,68,69,96,109]. Moreover, several studies conducted on animals [64,66], as well as humans [62,63,65], have reported the palpable advantages of using NS and TQ for periodontal disease prevention and treatment, besides the regular scaling and root planning, on clinical, molecular and histological levels. As explained in the current review, such periodontal health favoring effects of TQ and NS extracts seem to be encouraged through the distinct antibacterial, antioxidant/inflammatory and potentially regenerative mechanisms of these rich natural compounds. However, further detailed studies on NS and TQ-mediated periodontitis treatment are required at the cellular and molecular levels to investigate the exact mechanisms of action of NS and its constituents, as well as to provide an extended overview on the effects of these compounds in combinations with other periodontal-related therapies and medications. According to the current status of periodontal studies investigating TQ and NS, the authors of this review would recommend an intra-pocket TQ gel application (0.1–0.2%) as a natural adjunctive procedure to regular periodontal therapy. As topical administration of (0.1–0.2%) TQ has presented beneficial results of periodontal health in almost all available studies on in vivo (animal), in vitro and RCT levels (Table 3), this mode of TQ application seems to be the currently safest and most beneficial to periodontal patients. Overall, the reports that examined the role of *NS* and TQ are still initial and might need further elaboration and expansion, especially on RCT level, but the current outcomes revealed an extraordinary potential of future therapeutic integration of these natural compounds into the regular periodontal therapy.

## Figures and Tables

**Table 1:** Active compounds and elements isolated from *Nigella sativa* seeds and their percentages.

Compound and Elements	Percentages
Thymoquinone	30–48%
Thymohydroquinone, dithymoquinone, *p*-cymene	7–15%
Carvacrol	6–12%
4-Terpineol	2–7%
*t*-Anethol	1–4%
Longifolene (a sesquiterpene)	1–8%
Nigellicimine, nigellicimine-N-oxide, nigellidine, nigellicine, α-hederin, saponin, carvone, limonene, citronellol	<1% (trace amounts)
Minerals (calcium, potassium, magnesium, aluminum, copper, phosphorus, zinc, iron, manganese)	<1% (trace amounts)

**Table 2:** The potential thymoquinone-related toxicity and its reported effects.

Potential Thymoquinone-Related Toxicity	Reported Effects
Reproductive Toxicity	Elevated fetal resorption and serum amylase
Teratogenic Toxicity	Elevated DNA damage index and chromosomal aberrations
Acute Toxicity	Increased reactions with cellular nucleophiles and MDA with decreased GSH and GST
Subacute Toxicity	Elevated pyknotic nuclei and cell degeneration

Abbreviations: GSH: glutathione; GST: glutathione S-transferase; MDA: malondialdehyde.

**Table 3:** Main studies in relation to thymoquinone (TQ) and *Nigella sativa* (NS) of interest in periodontal disease.

Compound	Study Type	Sample Studied, n	Adminitration (Dosage, Frecuency and Duration)	Main Effects	Reference
TQ	RCT	Systemic healthy periodontitis male and female patients with at least 2 periodontally involved sites (≥5 mm), n = 20	0.2% TQ topical (intra-pocket) oral gel; repeated every week beginning from baseline up to 4 weeks.	Significant decrease in PPD, GCF-ALP levels and rise in CAL Antibacterial effect of TQ against against *P. gingivalis*, *A. actinomycetemcomitans* and *P. intermedia*	[62]
TQ	RCT	Systemic healthy chronic periodontitis male and female patients (25–58 years) with at least 10 periodontally involved sites (≥5 mm), n = 48	0.1% TQ topical (intra-pocket) oral gel; immediately after accomplishment of SRP. The gel was applied again after 48 h and the gel applied sites were covered with a periodontal pack for 7 days. Results were recorded at baseline and at weeks 4 and 12 after treatment.	Significant improvement in PI, GI, PPD, CAL Significant improvement in the levels of IL-1β and TAOC	[63]
TQ	RCT	Systemic healthy chronic periodontitis male patients (35–56 years) with at least 4 periodontally involved sites (≥5 mm), n = 12	Biodegradable chitosan subgingival periodontal chip with integrated 0.25 mg TQ with second chip insertion at day 14. Results were recorded at day 14 and 60 of the trial.	Significant gains in CAL	[65]
TQ	Animal	Male Wistar rats with ligature-induced periodontal inflammation (300 ± 10 g), n = 8	Systemic intragastric (10 mg/kg, daily for 11 days)	Significant reduction of alveolar bone loss and inflammatory cell infiltration Maintenance of osteoblastic activity	[66]
TQ	Animal	Male Fisher rats (21 days old), n = 16	0.2% TQ topical oral gel and systemic administration in drinking water. The oral gel was applied daily over gingiva and the drinking water was changed 3 times a week. Outcomes were measured at day 1, 7 and 35.	Significant reduction in BOP, PI in rats that received TQ as oral gel or systemically Less evident signs of gingivitis and periodontitis histologically in TQ treated rats Antibacterial effect of TQ against subgingival bacteria	[64]
TQ	In vitro	Bacterial strains of *Fusobacterium nucleatum* ATCC25586 (FN), *Actinomyces naeslundii* X600 (AN) and *Streptococcus mitis* ATCC 903 (*SM*)	0.1%, 0.01% and 0.05% TQ reagents were added to bacterial formed biofilms and incubated for 24 h and for 30 min	Inhibitory effect of 0.1% TQ on *FN*-containing biofilm Significantly decreased biofilm formation of *FN* pretreated with 0.01% TQ Significant cleansing effect of 0.01% and 0.05% TQ on *FN*-containing biofilm	[67]
TQ	In vitro	Bacterial strains of *Fusobacterium nucleatum* ATCC 25586 (FN) and *Porphyromonas gingivalis* A7436 (PG)	10% TQ reagent was added to bacterial formed biofilms and incubated for 48 h	The minimum inhibitory concentration (MIC) of TQ was 12.5 and 1.56 μg/mL in FN and PG, respectively. Sub-MIC concentrations of TQ prevented biofilm formation, hemolysis activities and H2S generation of *FN* and *PG* and TQ disintegrated bacterial membranes and reduced the expression of virulence factors in *FN* and *PG*	[68]
*NS*	In vitro	Twelve gram-positive and eleven Gram-negative bacterial strains, including *Staphylococcus aureus*, *Enterococcus faecalis, Staphylococcus epidermidis*, *Porphyromonas* sp., *Proteus* sp., *Streptococcus pneumoniae*, *Klebsiella pneumoniae*, *Acinetobacter baumannii/calcoaceticus* and *Veillonella* sp., isolated from supra- and subgingival plaque of periodontal patients	Methanol and essential oil solutions of *NS* 2% ±0.35 dry extract were added to the isolated bacteria in microdilution test and Agar well diffusion assay	Antibacterial effect of the essential oil and less efficiency of the methanol against all tested bacteria	[69]

PPD: Probing pocket depth; GCF-ALP: Gingival crevicular fluid alkaline phosphatase; CAL: Clinical attachment level; SRP: Scaling and root planning; PI: Plaque index; GI: Gingival index; TAOC: Total antioxidant capacity; FN: Fusobacterium nucleatum; AN: Actinomyces naeslundii; PG: Porphyromonas gingivalis.
